# Dermatoglyphic pattern, an anatomical indicator of bronchial asthma: A case control study

**DOI:** 10.6026/973206300214443

**Published:** 2025-12-15

**Authors:** Swapnesh Sagar, Vikas Rangare, Kapil Raghuwanshi, Sudhakar Petkar

**Affiliations:** 1 Department of Anatomy, Mansarovar Medical College & MGU Hospital, Sehore, Madhya Pradesh, India; 2 Department of Medicine, Chhindwara Institute of Medical Sciences, Chhindwara, Madhya Pradesh, India; 3 Department of Biochemistry, Chhindwara Institute of Medical Sciences, Chhindwara, Madhya Pradesh, India

**Keywords:** Dermatoglyphic patterns, anatomical marker, bronchial asthma

## Abstract

Dermatoglyphic patterns may be used to diagnose bronchial asthma, a genetically based chronic inflammatory airway disease. Therefore, it is of interest to compare patterns in asthma patients and a control group in order to assess dermatoglyphic pattern as a non-invasive marker for bronchial asthma. 102 asthma sufferers and 102 controls participated in the study, and the dermatoglyphic patterns of the two groups differed statistically significantly. The case group's whorl patterns were significantly higher than the control group's but their arch and loop patterns were smaller. Dermatoglyphics, particularly whorl patterns, can be used as a non-invasive anatomical marker and could be helpful in diagnosing bronchial asthma, according to the results.

## Background:

Millions of people worldwide suffer with bronchial asthma, a chronic inflammatory airway illness that is typified by bronchial tree hyperactivity and bronchoconstriction [1]. In developed nations, it is now among the most prevalent chronic illnesses affecting kids and teenagers [2]. Asthma is a polygenic and complex disease, meaning that a variety of variables have a role in its development. The polygenic and multifactorial development of asthma is influenced by both environmental and genetic factors. Environmental variables both cause and affect the development of disease, and genetic predisposition is thought to be significant [3, 4]. Dermatoglyphics is a scientific technique for interpreting palm ridges and lines [5]. The distinctive, permanent, universal, and inimitable patterns that are genetically determined are characteristics of dermatoglyphic patterns. These patterns are essential to medical genetics and criminal investigations because they reappear even after destruction. Environmental influences throughout the early stages of pregnancy can have an impact on these genetically regulated patterns. During the first three months of pregnancy, environmental influences may have an impact on the dermatoglyphic features, which are genetically controlled [6]. Dermatoglyphics can be used to identify people who have a genetic predisposition to asthma and other conditions, but it is not a diagnostic tool. The relevance of dermatoglyphics is not diagnosis but prognosis; it is not the description of an existing ailment. Rather, it is the identification of individuals who have a hereditary susceptibility to the development of specific diseases [7]. Dermatoglyphics has been investigated in relation to a number of clinical conditions that are linked to developmental and chromosomal abnormalities, such as ischemic heart disease, schizophrenia, diabetes mellitus, cardiovascular disease, and Turner's syndrome [8, 9]. Recent work has shown that women with Turner syndrome exhibit significantly altered frequencies of arches, ulnar loops, whorls, and palmar creases compared with women with normal karyotype [10]. Fogle T. [11] found that ulnar loops on index finger and radial loops on ring finger were mostly seen in patients with down syndrome. Ridge patterns are impacted by abnormal chromosomal complements, such as in Down syndrome, which might be useful for diagnosis. Gupta *et al.* [12] in their innovative researcher had shown a high correlation between ridge pattern and bronchial asthma. Fingerprint patterns fall into three main categories: Whorls (24-34%), Loops (61-71%), and Arches (4-5%). Ridges that continue from side to side without turning back are called arches. There are two types: tented arches, which have ridges that rise sharply, and simple arches. Loops: A line from delta to core is touched or crossed by one or more ridges as they enter and recurve, ending on the same side. Depending on the direction of opening, they are categorized as either radial or ulnar loops. Whorls: Ridges with two or more deltas form at least one circuit. Concentric, spiral, mixed, central pocket, twin, and accidental whorls are among the types. Ridge courses can be single or double circuits that round the core [13]. Dermatoglyphics is becoming more popular as a genetic marker since genes affect dermal ridges, which don't change throughout the course of a person's life. For screening and early identification of people at risk, dermatoglyphics is a non-invasive, economical, and time-efficient method. Therefore, it is of interest to compares dermatoglyphics in patients with bronchial asthma to the control group in order to evaluate dermatoglyphics as an anatomical marker for bronchial asthma that is non-invasive, and identify a particular dermatoglyphic pattern in bronchial asthma patients that may be useful for diagnosis.

## Materials and Methods:

From March to August 2019, 102 patients with a clinical diagnosis of bronchial asthma who visited the T.B. and Chest OPD of M.G.M. Medical College and M.Y. Hospital in Indore (M.P.) participated in the current study. As controls, 102 healthy, normal people were chosen. The present investigation employed the Cummins and Midlo ink method for dermatoglyphic recording [14]. All participants gave their informed consent. ethical clearance obtained from the relevant authority. The hands were carefully cleaned with soap and dried before the finger prints were taken. Recording was done with the palm and fingers over the inkpad. Fingerprints were examined under a magnifying glass to determine the pattern type and other factors.

## Inclusion criteria:

Patients who were 18 years of age or older and had a clinical diagnosis of bronchial asthma were included in the case group, while healthy individuals who were matched for age and gender were included in the control group.

## Exclusion criteria:

Exclusion criteria included being under the age of eighteen, having polydactyly or distorted ridges and lines, such as from burns, and refusing to take part in the study.

## Analysis of statistics:

The proportional or frequency distribution of the characteristics was used to produce descriptive statistics. Both qualitative and quantitative data categories underwent frequency distribution analysis. Overall, it was presumed that the collected data was normal and that the observations made for continuous variables had followed a normal distribution. The significant difference between the mean values of the various numerical parameters of the two groups was determined using the Student t test for two sample means. To ascertain whether two variables were associated, the Chi Square Test was utilized. Using the chi square test, the various parameter distributions were linked to various morbidities. The significance level was defined as the P Value < 0.05.

## Results:

The investigation found no appreciable difference between the mean age of the patients and controls. There were notable differences between the patients and controls in the arch, loop, and whorl patterns. Table 1 (see PDF) demonstrates that whereas the whorl pattern was considerably greater in the case group, the arch and loop patterns were significantly higher in the control group. In the case group, the right-hand whorl pattern was noticeably higher as shown in Table 2 (see PDF). In the case group, the left-hand whorl pattern was substantially larger as mentioned in Table 3 (see PDF). In the case group, the thumb's whorl pattern was considerably higher as revealed in Table 4 (see PDF). Compared to the control group, the case group's whorl pattern at the index finger was noticeably higher as exhibited in Table 5 (see PDF). In this study, patients' and controls' middle, ring, and little finger patterns were examined. The results showed that while the arch pattern was statistically non-significant at the middle finger, the loop and whorl patterns were significant as appeared in Table 6 (see PDF). The ring finger whorl pattern was significantly greater in the case group, which exhibited a less prominent arch pattern as indicated in Table 7 (see PDF). Little finger patterns in the case and control groups were substantially different; the former had a higher whorl pattern and a smaller loop pattern as expressed in Table 8 (see PDF). Therefore, this research indicates that an excess of whorl patterns is associated with bronchial asthma.

## Discussion:

Personal identification marks, or dermatoglyphics, are now a useful tool in genetic, medical, and anthropological research. According to Xue *et al.* [15] there may be a genetic foundation for the association between ADAM33 polymorphisms and asthma. The intent of this study was to determine the association between individuals with bronchial asthma and dermatoglyphic changes in the tips of their fingers. The case and control group's average age were 45.56 + 12.25 and 45.42 + 13.06 years, respectively. With fewer loop patterns in the fourth and fifth fingers of cases (26.47% and 53.43%) than in the fifth and sixth fingers of controls (53.43% and 73.04%), the study discovered that loop patterns were substantially lower in cases than in controls. The results of our investigation are in line with Pakhale *et al.* [16] who discovered that individuals with bronchial asthma had less loop patterns than healthy people. When comparing asthma cases to controls, Sagar *et al.* [17] discovered a noticeably a smaller number of arch patterns. Arch patterns were also found to be considerably smaller in cases compared to healthy individuals, with a notable difference in the fourth digit. The other digits' arch pattern was determined to be non-significant, indicating that arch pattern is not crucial for the early identification of instances of bronchial asthma. According to the study, patients had substantially more whorl patterns than controls. All of the finger digits showed patterns, but the first digit (46.57%) of cases had the highest frequency compared to the control (24.94%). This was comparable to the findings of Sreenivasulu *et al.* [18] who found that the first digit of cases had a significantly higher frequency of whorls patterns. As was also noted in our study, Rao *et al.* [19] discovered noticeably larger whorls in the third and fourth digits of cases. In our investigation, the majority of whorl patterns were observed in the majority of case numbers when compared to the control. The results were comparable to those of Mahajan *et al.* [20] According to our research, there are substantially more whorl patterns overall in all bronchial asthma cases, which may be a crucial pattern for early case detection. According to our study's findings, dermatoglyphic patterns may be useful in diagnosing bronchial asthma and in predicting a person's risk of developing the illness.

## Conclusion:

The study's findings led us to the following conclusion: (a) Dermatoglyphics are non-invasive and cost-effective anatomical markers to detect asthma because they have a strong link with these patients, (b) among dermatoglyphic patterns, whorls were more common in bronchial asthma cases, with most of them present in all of the patients' fingers, which may help to anticipate its occurrence. The theory that bronchial asthma sufferers had higher whorl patterns than healthy people was validated.
